# Supplementary material for: Clinical Associations between Serial Electrocardiography Measurements and Sudden Cardiac Death in Patients with End-Stage Renal Disease Undergoing Hemodialysis
Source: J Clin Med. 2021 Apr 29;10(9):1933. doi: 10.3390/jcm10091933 (PMC8124551; doi:10.3390/jcm10091933)
Supplement: Supplementary file 1 [file jcm-10-01933-s001.zip › jcm-1173031-supplementary.pdf]

Clinical associations between serial electrocardiography measurements and sudden cardiac death in patients with end-stage renal disease undergoing hemodialysis: Multi-center, retrospective cohort study

Hyun Jin Lee [1,†], A Reum Choe [2,†], HaeJu Lee [3], Dong Ryeol Ryu [2], Ea Wha Kang [4], Jung Tak Park [5], Su Hwan Lee [6,*,‡] and Junbeom Park [7,*,‡]

Supplementary Materials

Table S1. ROC analysis of the pre-HD QT peak-end interval at all leads as predictors of SCD

Table 2. Baseline characteristics of survivors and SCD patients after propensity score matching for age and sex.

Table S3. Pre-hemodialysis ECG findings after propensity score matching for age and sex

Table S4. Post-hemodialysis ECG findings after propensity score matching for age and sex

Table S5. Baseline characteristics of survivors and cardiovascular disease patients

Table S6. Pre-hemodialysis ECG findings of survivors and cardiovascular disease patients

Table S7. Post-hemodialysis ECG findings of survivors and cardiovascular disease patients

Table S8. Changes in ECG parameters after HD (ECG before HD – ECG after HD)

Table S9. Baseline characteristics of the patients who older than 12 months after HD

Table S10. Pre-hemodialysis ECG findings of the patients who older than 12 months after HD.

Table S11. Post-hemodialysis ECG findings of the patients who older than 12 months after HD.

Table S12. Changes in ECG parameters after HD (ECG before HD – ECG after HD) in patients who older than 12 months after HD.

Figure S1. Comparison of ECG changes before and after HD in survivors and SCD patients using a linear mixing model

Figure S2. Comparison of ECG changes before and after HD in survivors and CVD death patients using a linear mixing model

Table S1. ROC analysis of the pre-HD QT peak-end interval at all leads as predictors of SCD.

QT peak-end interval (ms)	AUC	95% confidence interval
II	0.771	0.656–0.886
III	0.759	0.635–0.883
aVF	0.75	0.618–0.883
V1	0.8	0.695–0.906
V2	0.83	0.741–0.919
V3	0.809	0.699–0.919
V4	0.801	0.693–0.910
V5	0.763	0.636–0.890
V6	0.768	0.642–0.894

HD, hemodialysis; SCD, sudden cardiac death; ROC, receiver operating characteristic; AUC, area under the curve.

Table S2. Baseline characteristics of survivors and SCD patients after propensity score matching for age and sex.

	Survivor (*n* = 195)	SCD (*n* = 39)	*p*-Value
Age (years)	67.4 ± 10.8	69.2 ± 12.5	0.348
Male sex (*n*, %)	123 (63.1)	25 (64.1)	0.903
Body surface area (m^2)	1.67 ± 0.2	1.66 ± 0.22	0.877
Body mass index (kg/m^2)	23.4 ± 3.7	22.8 ± 3.9	0.382
Hypertension (*n*, %)	180 (92.3)	33 (84.6)	0.387
Diabetes mellitus (*n*, %)	120 (61.5)	30 (76.9)	0.062
Duration of HD (months)	59.4 ± 49.2	54.9 ± 52.8	0.611
Echocardiography before HD			
Pre-HD LVEF (%)	58.3 ± 12.0	54.8 ± 12.4	0.153
Pre-HD LVEDD (mm)	52.3 ± 6.4	53.9 ± 10.1	0.333
Pre-HD LVESD (mm)	35.9 ± 7.4	38.1 ± 11.2	0.415
Pre-HD E/E'	16.4 ± 7.1	19.4 ± 7.7	0.071
Echocardiography after HD			
Post-HD LVEF (%)	58.0 ± 12.3	46 ± 11.8	<0.001
Post-HD LVEDD (mm)	51.5 ± 6.0	52.7 ± 10.5	0.591
Post-HD LVESD (mm)	35.9 ± 7.7	39.9 ± 10.7	0.093
Post-HD E/E'	17.8 ± 7.6	22.9 ± 9.4	0.037
Laboratory findings after HD			
Hb (g/dL)	10.5 ± 1.4	9.3 ± 1.5	<0.001
Hct (%)	32.9 ± 10.2	28.2 ± 4.8	0.049
BUN (mg/dL)	52.2 ± 22.2	58.5 ± 30.7	0.401
Cr (mg/dL)	8.1 ± 3.0	6.8 ± 3.7	0.096
eGFR (EPI)	6.6 ± 4.4	10.0 ± 8.4	0.102
Sodium (mEq/L)	136.4 ± 13.3	137.6 ± 5.4	0.691
Potassium (mEq/L)	4.8 ± 1.3	4.8 ± 1.3	0.821
T.Calcium (mg/dL)	8.8 ± 1.1	8.6 ± 0.7	0.635

Values are expressed as *n* (%) or means (standard deviations) unless otherwise indicated; SCD, sudden cardiac death; HD, hemodialysis; LV, left ventricle; EF, ejected fraction; LVEDD, left ventricle end-diastolic dimension; LVESD, left ventricle end-systolic dimension; E/E′, E: mitral peak velocity of early filling, E′: early diastolic mitral annular velocity; Hb, hemoglobin; Hct, hematocrit; BUN, blood urea nitrogen; Cr, creatinine; eGFR, estimated glomerular filtration rate; T.Calcium, Total calcium .

Table S3. Pre-hemodialysis ECG findings after propensity score matching for age and sex.

	Survivor (*n* = 195)	SCD (*n* = 39)	*p*-Value
Atrial fibrillation (*n*, %)	3 (1.5)	3 (7.7)	0.054
Heart rate (/min)	76.1 ± 14.6	80.6 ± 16.7	0.16
PR interval (ms)	170.0 ± 29.8	175.2 ± 37.9	0.549
QRS duration (ms)	94.5 ± 15.6	100.6 ± 14.9	0.107
QT interval (ms)	403.4 ± 51.6	395.6 ± 49.0	0.469
QTc interval (ms)	450.7 ± 28.3	456.2 ± 29.9	0.357
Dispersion of QT (ms)	56.4 ± 35.4	67.9 ± 22.7	0.105

QT peak-end interval (ms)			
II	108.2 ± 47.4	183.9 ± 67.4	<0.001
III	106.6 ± 82.8	174.8 ± 72.3	<0.001
aVF	101.6 ± 45.4	178.4 ± 70.8	<0.001
V1	105.5 ± 47.5	192.5 ± 70.5	<0.001
V2	116.7 ± 46.9	206.8 ± 67.7	<0.001
V3	120.5 ± 49.4	209.7 ± 75.9	<0.001
V4	115.1 ± 51.5	201.8 ± 71.8	<0.001
V5	115.3 ± 50.7	202.1 ± 76.7	<0.001
V6	113.3 ± 52.2	200.3 ± 80.8	<0.001

Values are expressed as *n* (%) or means (standard deviations); SCD, sudden cardiac death; ECG, echocardiography.

Table S4. Post-hemodialysis ECG findings after propensity score matching for age and sex.

	**Survivor (*n* = 195)**	**SCD (*n* = 39)**	***p*-Value**
Atrial fibrillation (*n*, %)	7 (3.6)	3 (7.7)	0.205
Heart rate (/min)	81.4 ± 15.1	89.8 ± 22.3	0.035
PR interval (ms)	174.3 ± 33.7	151.0 ± 44.6	0.019
QRS duration (ms)	96.9 ± 32.8	105.6 ± 33.0	0.281
QT interval (ms)	408.7 ± 56.1	396.9 ± 62.7	0.31
QTc interval (ms)	455.5 ± 52.6	464.1 ± 47.3	0.376
Dispersion of QT (ms)	53.6 ± 22.4	66.6 ± 43.8	0.115
QT peak-end interval (ms)			
II	88.2 ± 20.7	83.3 ± 37.1	0.486
III	86.9 ± 67.0	81.5 ± 35.3	0.66
aVF	82.9 ± 20.0	78.4 ± 31.9	0.457
V1	89.3 ± 23.3	92.0 ± 22.0	0.556
V2	104.3 ± 68.8	90.4 ± 21.4	0.301
V3	103.8 ± 20.6	94.2 ± 24.8	0.019
V4	101.8 ± 29.9	93.7 ± 28.3	0.192
V5	96.3 ± 21.6	86.0 ± 22.2	0.02
V6	91.8 ± 20.6	91.3 ± 25.8	0.912

Values are expressed as *n* (%) or means (standard deviations); SCD, sudden cardiac death; ECG, echocardiography.

Table S5. Baseline characteristics of survivors and cardiovascular disease patients.

	**Survivor (*n* = 387)**	**CVD (*n* = 71)**	***p*-Value**
Age (years)	59.8 ± 13.7	70.5 ± 11.7	<0.001
Male sex (*n*, %)	189 (48.8)	39 (54.9)	0.345
Body surface area (m^2)	1.64 ± 0.19	1.61 ± 0.22	0.211
Body mass index (kg/m^2)	23.0 ± 3.7	22.1 ± 3.7	0.089
Hypertension (*n*, %)	349 (90.2)	64 (90.1)	0.884
Diabetes mellitus (*n*, %)	206 (53.2)	49 (69)	0.011
Duration of HD (months)	64.5 ± 53.0	45.9 ± 52.4	0.008
Duration of HD (months, median [interquartile range])	58.6 (30.4-79.1)	32 (9.2-57.4)	<0.001
Echocardiography before HD			
Pre-HD LVEF (%)	59.4 ± 11.3	52.7 ± 14.5	0.002
Pre-HD LVEDD (mm)	51.9 ± 6.6	53.2 ± 9.2	0.365
Pre-HD LVESD (mm)	35.5 ± 7.3	37.9 ± 10.6	0.171
Pre-HD E/E'	15.6 ± 6.6	18.1 ± 8.1	0.02
Echocardiography after HD			
Post-HD LVEF (%)	58.4 ± 11.6	44.4 ± 13.9	<0.001
Post-HD LVEDD (mm)	51.2 ± 6.0	52.2 ± 10.2	0.495
Post-HD LVESD (mm)	35.3 ± 7.1	39.8 ± 10.8	0.012

Post-HD E/E'	17.2 ± 7.9	20.1 ± 7.3	0.079
Laboratory findings after HD			
Hb (g/dL)	10.5 ± 1.4	9.4 ± 2.0	0.004
Hct (%)	32.6 ± 7.8	28.4 ± 6.3	0.005
BUN (mg/dL)	54.8 ± 21.9	57.1 ± 29.0	0.681
Cr (mg/dL)	8.4 ± 3.2	5.9 ± 3.3	<0.001
eGFR (EPI)	6.5 ± 5.6	11.2 ± 8.1	<0.001
Sodium (mEq/L)	137 ± 9.8	138.1 ± 5.4	0.529
Potassium (mEq/L)	4.9 ± 0.8	4.8 ± 1.2	0.441
T.Calcium (mg/dL)	8.9 ± 1.0	8.7 ± 0.9	0.477

Values are expressed as *n* (%) or means (standard deviations) unless otherwise indicated; CVD, cardiovascular disease; HD, hemodialysis; LV, left ventricle; EF, ejected fraction; LVEDD, left ventricle end-diastolic dimension; LVESD, Left ventricle end-systolic dimension; E/E′, E: mitral peak velocity of early filling, E′: early diastolic mitral annular velocity; Hb, hemoglobin; Hct, hematocrit; BUN, blood urea nitrogen; Cr, creatinine; eGFR, estimated glomerular filtration rate; T.Calcium, Total calcium.

Table S6. Pre-hemodialysis ECG findings of survivors and cardiovascular disease patients.

	**Survivor (*n* = 387)**	**CVD (*n* = 71)**	***p*-Value**
Atrial fibrillation (*n*, %)	5 (1.3)	5 (7.0)	0.01
Heart rate (/min)	76.2 ± 14.6	80.0 ± 14.2	0.086
PR interval (ms)	168.9 ± 27.6	170.1 ± 31.8	0.81
QRS duration (ms)	92.6 ± 14.0	101.6 ± 20.3	0.01
QT interval (ms)	405.5 ± 47.9	400.9 ± 70.8	0.569
QTc interval (ms)	451.8 ± 28.8	466.9 ± 33.7	0.001
Dispersion of QT (ms)	55.5 ± 32.4	72.8 ± 29.0	0.001
Dispersion of QTc (ms)			
QT Peak-end interval (ms)			
II	113.4 ± 50.0	154.2 ± 70.8	<0.001
III	110.0 ± 69.2	141.6 ± 77.3	0.001
aVF	108.4 ± 48.4	145.9 ± 75.7	0.001
V1	112.0 ± 49.6	154.9 ± 80.3	0.001
V2	123.9 ± 51.5	169.1 ± 78.0	<0.001
V3	126.3 ± 53.3	170.6 ± 84.8	0.001
V4	122.2 ± 54.5	166.2 ± 78.0	<0.001
V5	121.1 ± 53.6	164.8 ± 81.6	0.001
V6	118.5 ± 54.4	161.3 ± 84.1	0.001

Values are expressed as *n* (%) or means (standard deviations); CVD, cardiovascular disease; ECG, echocardiography.

Table S7. Post-hemodialysis ECG findings of survivors and cardiovascular disease patients.

	**Survivor (*n* = 387)**	**CVD (*n* = 71)**	***p*-Value**
Atrial fibrillation (*n*, %)	12 (3.1)	12 (16.9)	<0.001
Heart rate (/min)	81.9 ± 15.6	92.9 ± 27.5	0.016
PR interval (ms)	171.2 ± 31.8	150.1 ± 42.3	0.017
QRS duration (ms)	95.2 ± 26.1	109.0 ± 30.8	0.011
QT interval (ms)	409 ± 51.4	394.7 ± 67.6	0.137
QTc interval (ms)	459.6 ± 46.8	468.0 ± 50.4	0.248
Dispersion of QT (ms)	52.0 ± 21.0	69.6 ± 39.2	0.003
Dispersion of QTc (ms)	62.1 ± 23.1	78.9 ± 51.4	0.082
QT peak-end interval (ms)			
II	89.1 ± 37.0	92.0 ± 45.7	0.666
III	84.2 ± 52.5	86.3 ± 48.9	0.788
aVF	83.3 ± 20.3	87.3 ± 50.2	0.575
V1	88.3 ± 22.9	93.1 ± 37.4	0.393

V2	102.1 ± 50.5	94.5 ± 36.8	0.325
V3	102.0 ± 20.0	96.0 ± 37.7	0.275
V4	98.4 ± 26.8	99.2 ± 40.3	0.901
V5	93.5 ± 21.9	94.4 ± 41.5	0.894
V6	90.0 ± 20.5	97.4 ± 42.8	0.246

Values are expressed as *n* (%) or means (standard deviations); CVD, cardiovascular disease; ECG, echocardiography.

Table S8. Changes in ECG parameters after HD (ECG before HD – ECG after HD).

	**Survivor (*n* = 387)**	**CVD (*n* = 71)**	***p*-Value**
QT interval (ms)	−6 (−36, 26)	26 (−50, 90.5)	0.206
QTc interval (ms)	−8.5 (−32, 12)	3.5 (−46.8, 56.5)	0.224
Dispersion of QT (ms)	4.3 (−13.9, 27.8)	28 (−14.5, 48)	0.018
QT peak-end interval (ms)			
II	8 (−9.4, −71.2)	45 (5, 91)	0.001
III	12 (−8, 69.4)	36 (−11, 88)	0.011
aVF	12 (−8, 61.8)	19 (−7, 123)	0.007
V1	8 (−12, 64.4)	24 (−6, 132)	0.006
V2	12 (−8, 63)	28 (4, 138)	<0.001
V3	8.7 (−8, 70.2)	38 (−3, 190)	<0.001
V4	8 (−10, 64.7)	28 (−8, 131)	0.01
V5	12 (−9.2, 96.6)	32 (−7, 156)	0.005
V6	12 (−4.2, 66.2)	24 (−8, 149)	0.052

Values are expressed as medians (interquartile range); CVD, cardiovascular disease; ECG, echocardiography.

Table S9. Baseline characteristics of the patients who older than 12 months after HD.

**Variable**	**Survivor (*n* = 329)**	**SCD (*n* = 31)**	***p*-Value**
Age (years)	59.6 ± 13.7	66.9 ± 12.3	0.004
Male (*n*, %)	159 (48.3)	22 (71.0)	0.016
Body surface area (m2)	1.64 ± 0.19	1.70 ± 0.21	0.097
Body mass index (kg/m2)	23.0 ± 3.6	23.1 ± 4.1	0.878
Hypertension (*n*, %)	298 (90.6)	27 (87.1)	0.532
Diabetes mellitus (*n*, %)	178 (54.1)	25 (80.6)	0.004
Duration of HD (months)	71.6 ± 51.8	64.9 ± 51.9	0.491
Echocardiography before HD			
Pre-HD LVEF (%)	59.4 ± 10.6	54.1 ± 12.1	0.022
Pre-HD LVEDD (mm)	52.0 ± 6.2	54.1 ± 9.4	0.359
Pre-HD LVESD (mm)	35.5 ± 6.7	38.8 ± 11.2	0.269
Pre-HD E/E'	16.0 ± 6.9	20.0 ± 8.1	0.018
Echocardiography after HD			
Post-HD- HD LVEF (%)	58.5 ± 11.5	46.8 ± 11.9	<0.001
Post-HD LVEDD (mm)	51.2 ± 5.7	52.2 ± 10.7	0.686
Post-HD LVESD (mm)	35.2 ± 6.8	39.9 ± 10.9	0.064
Post-HD E/E'	17.2 ± 7.6	22.9 ± 9.4	0.016
Laboratory findings after HD			
Hb (g/dL)	10.6 ± 1.5	9.5 ± 1.5	0.006
Hct (%)	32.8 ± 8.3	28.9 ± 4.9	0.078
BUN (mg/dL)	54.2 ± 22.2	57.2 ± 30.1	0.632
Cr (mg/dL)	8.4 ± 3.2	7.5 ± 3.8	0.267
eGFR (EPI)	6.5 ± 6.0	9.9 ± 9.5	0.194
Sodium (mEq/L)	136.7 ± 10.5	136.8 ± 4.9	0.98
Potassium (mEq/L)	4.9 ± 0.8	4.6 ± 0.9	0.13
T.Calcium (mg/dL)	8.9 ± 1.0	8.7 ± 0.6	0.502

Table S10. Pre-hemodialysis ECG findings of the patients who older than 12 months after HD.

Variable	Survivor (*n* = 329)	SCD (*n* = 31)	*p*-Value
Atrial fibrillation (*n*, %)	4 (1.7)	3 (13)	0.016
Heart rate (/min)	75.4 ± 14.2	78.2 ± 14.5	0.37
PR interval (ms)	168.2 ± 27.4	175.7 ± 36.7	0.351
QRS duration (ms)	92.6 ± 14.1	99.0 ± 14.5	0.082
QT interval (ms)	407.0 ± 47.3	397.1 ± 48.5	0.339
QTc interval (ms)	452.0 ± 28.7	453.0 ± 28.9	0.872
Dispersion of QT (ms)	56.6 ± 33.2	67.2 ± 23.0	0.135
QT Peak-end interval (ms)			
II	116.5 ± 51.3	181.2 ± 72.4	<0.001
III	110.2 ± 51.3	171.2 ± 77.4	0.001
aVF	111.4 ± 48.7	175.0 ± 75.8	0.001
V1	114.9 ± 50.6	190.0 ± 74.5	<0.001
V2	126.3 ± 53.2	204.7 ± 71.4	<0.001
V3	129.6 ± 54.8	207.9 ± 79.9	<0.001
V4	125.0 ± 56.5	199.6 ± 77.4	<0.001
V5	124.1 ± 55.0	197.1 ± 82.1	<0.001
V6	121.7 ± 55.9	194.3 ± 86.1	0.001

Table S11. Post-hemodialysis ECG findings of the patients who older than 12 months after HD.

Variable	Survivor (*n* = 329)	SCD (*n* = 31)	*p*-Value
Atrial fibrillation (*n*, %)	10 (3.0)	3 (9.7)	0.138
Heart rate (/min)	81.7 ± 15.4	89.4 ± 20.7	0.058
PR interval (ms)	168.5 ± 31.6	158.6 ± 45.1	0.487
QRS duration (ms)	96.0 ± 27.6	102.3 ± 22.5	0.379
QT interval (ms)	409.6 ± 52.9	403.4 ± 60.5	0.555
QTc interval (ms)	459.5 ± 48.9	467.3 ± 47.4	0.418
Dispersion of QT (ms)	51.7 ± 20.9	68.7 ± 47.4	0.087
QT Peak-end interval (ms)			
II	89.2 ± 39.1	83.6 ± 37.7	0.483
III	84.6 ± 55.9	80.9 ± 38.3	0.744
aVF	83.2 ± 19.5	76.8 ± 31.1	0.327
V1	88.5 ± 23.1	92.9 ± 21.2	0.372
V2	102.4 ± 53.9	92.9 ± 21.4	0.408
V3	101.8 ± 20.1	92.6 ± 25.4	0.087
V4	97.5 ± 22.0	95.7 ± 25.6	0.714
V5	93.2 ± 22.1	88.1 ± 21.1	0.277
V6	89.0 ± 20.0	91.9 ± 24.5	0.585

Table S12. Changes in ECG parameters after HD (ECG before HD – ECG after HD) in patients who older than 12 months after HD.

Variable	Survivor (*n* = 329)	SCD (*n* = 31)	*p*-Value
QT interval (ms)	−6 (−35, 25)	44 (−8, 85)	0.996
QTc interval (ms)	−10 (−32.5, 14)	1 (−14.3, 44.5)	0.989
Dispersion of QT (ms)	6 (−12, 28.5)	31 (−1.5, 47.5)	0.006
QT Peak-end interval (ms)			
II	12 (−8.6, −78.6)	114 (85, 132)	<0.001

III	12 (−6.3, 77)	108 (770, 142)	<0.001
aVF	12 (−8, 65.6)	134 (120, 143)	<0.001
V1	8 (−12, 74.9)	142 (117, 151)	<0.001
V2	13.8 (−8, 79.8)	144 (108, 170)	<0.001
V3	12 (−8, 80.3)	154 (138, 191)	<0.001
V4	8 (−9.3, 69.2)	142 (95, 166)	<0.001
V5	16 (−7, 82.6)	160 (141, 178)	<0.001
V6	16 (−4, 74.9)	156 (92, 175)	<0.001

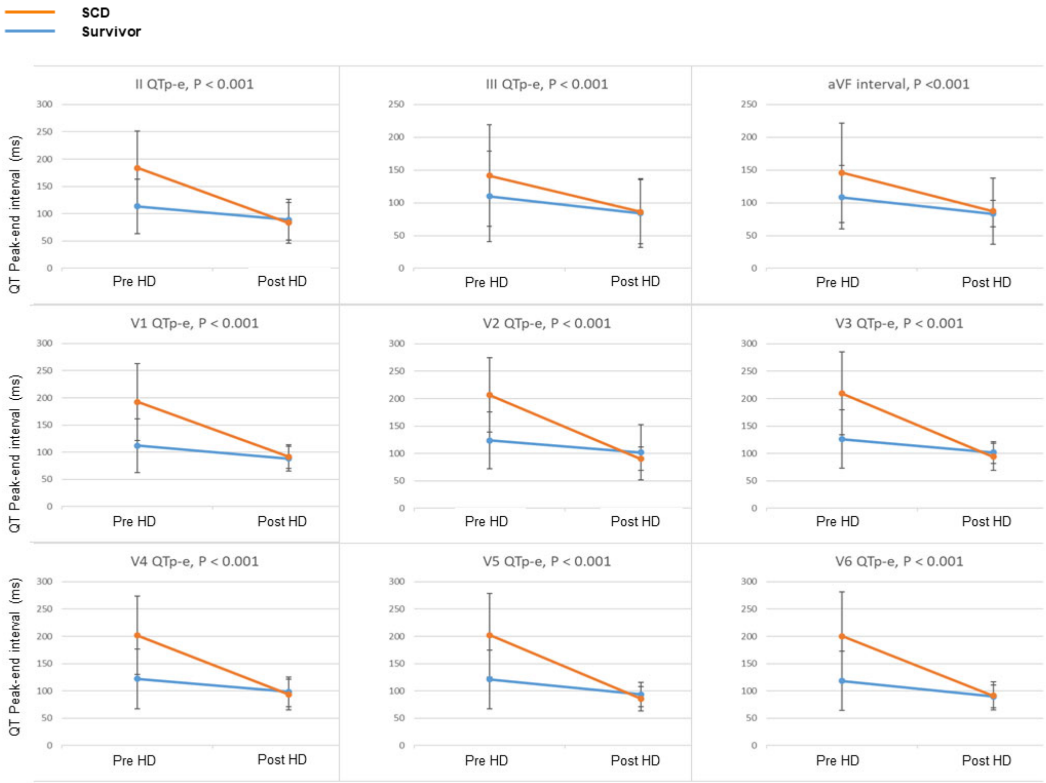

Figure S1. Comparison of ECG changes before and after HD in survivors and SCD patients using a linear mixing model. The change in QTpe interval before and after HD in the SCD group and the change in QTpe interval before and after HD in the survivor group were statistically significant. The SCD group showed a significantly larger change in the QTpe intervals of II, III, aVF, V1-V6 leads after HD than the survivor group

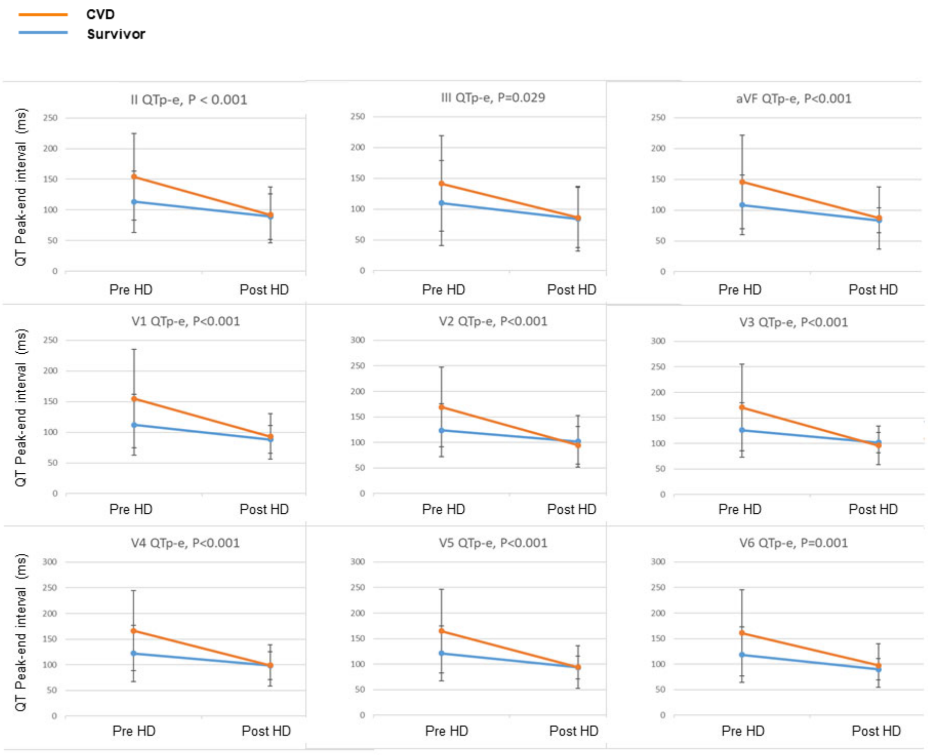

Figure 2. Comparison of ECG changes before and after HD in survivors and CVD death patients using a linear mixing model. The changes in ECG parameters (II, III, aVF, V1-V6 leads) after HD were significantly greater in the CVD mortality group than in the survivor group.
